# Effect of rapamycin on bone mass and strength in the α2(I)‐G610C mouse model of osteogenesis imperfecta

**DOI:** 10.1111/jcmm.14072

**Published:** 2018-12-30

**Authors:** John F. Bateman, Lisa Sampurno, Antonio Maurizi, Shireen R. Lamandé, Natalie A. Sims, Tegan L. Cheng, Aaron Schindeler, David G. Little

**Affiliations:** ^1^ Murdoch Children's Research Institute Parkville Victoria Australia; ^2^ Department of Biochemistry and Molecular Biology University of Melbourne Parkville Victoria Australia; ^3^ Department of Paediatrics University of Melbourne Parkville Victoria Australia; ^4^ Department of Biotechnological and Applied Clinical Sciences University of L'Aquila L'Aquila Italy; ^5^ St. Vincent's Institute of Medical Research Fitzroy Victoria Australia; ^6^ Department of Medicine at St. Vincent's Hospital The University of Melbourne Fitzroy Victoria Australia; ^7^ Orthopaedic Research and Biotechnology Unit The Children's Hospital at Westmead Sydney New South Wales Australia

**Keywords:** autophagy stimulation, biomechanics, diseases and disorders of bone, micro‐computed tomography, osteogenesis imperfecta, rapamycin treatment

## Abstract

Osteogenesis imperfecta (OI) is commonly caused by heterozygous type I collagen structural mutations that disturb triple helix folding and integrity. This mutant‐containing misfolded collagen accumulates in the endoplasmic reticulum (ER) and induces a form of ER stress associated with negative effects on osteoblast differentiation and maturation. Therapeutic induction of autophagy to degrade the mutant collagens could therefore be useful in ameliorating the ER stress and deleterious downstream consequences. To test this, we treated a mouse model of mild to moderate OI (α2(I) G610C) with dietary rapamycin from 3 to 8 weeks of age and effects on bone mass and mechanical properties were determined. OI bone mass and mechanics were, as previously reported, compromised compared to WT. While rapamycin treatment improved the trabecular parameters of WT and OI bones, the biomechanical deficits of OI bones were not rescued. Importantly, we show that rapamycin treatment suppressed the longitudinal and transverse growth of OI, but not WT, long bones. Our work demonstrates that dietary rapamycin offers no clinical benefit in this OI model and furthermore, the impact of rapamycin on OI bone growth could exacerbate the clinical consequences during periods of active bone growth in patients with OI caused by collagen misfolding mutations.

## 1 INTRODUCTION

Osteogenesis imperfecta (OI) is a serious inherited “brittle bone” disease most commonly resulting from mutations that compromise the function of type I collagen, either by reducing expression of *COL1A1* or *COL1A2*, or causing mutant protein misfolding and degradation.^1^ *COL1A1* and *COL1A2* encode the two collagen α‐chain subunits, α1(I) and α2(I), which heterotrimerize into the functional [α1(I)]_2_α2(I) collagen molecules. While *COL1A1* and *COL1A2* mutations are by far the most common cause (>85%), the genetic disease spectrum also encompasses mutations in components of the collagen folding and assembly machinery (*CRTAP*,* LEPRE1*,* PPIB, FKBP10*,* SERPINH1*), genes involved in bone formation (*SP7*), bone cell signalling genes (*SERPINF1*,* WNT1*), a cation‐specific channel (*TMEM38B*), and *IFITM5*. Precise genotype‐phenotype correlations are not fully established, but several generalizations can be made. Mutations that reduce *COL1A1* and *COL1A2* expression, such as nonsense and frameshift mutations that introduce premature termination codons and lead to nonsense‐mediated mRNA decay and haploinsufficiency, result in a milder clinical phenotype (OI type I). Mutations that introduce structural missense mutations into the collagen α‐chains result in more severe OI phenotypes (OI type II, III, IV). Of the many hundred missense mutations described, the most common mutations are glycine substitutions in triple helical domains of the α1(I) and α2(I) chains (>80%).^1^, ^2^ These substitutions interrupt the obligatory Gly‐X‐Y repeat sequence of the collagen helix, causing misfolding and a structurally abnormal helix.

The molecular mechanism of how these structural mutations cause the bone phenotype has long been thought to be because of a dominant negative effect of incorporating mutant collagen into the collagen trimers. This can affect collagen folding and reduce secretion, with even small amounts of secreted mutant‐containing trimers adversely affecting collagen fibril assembly, stability, and crucial collagen‐ECM interactions.^2^, ^3^ While the central tenets of this model are correct, recent studies on collagen I mutations in OI and collagen II and collagen X mutations in other skeletal dysplasias have added complexity to this model. Notably, the endoplasmic reticulum (ER) stress response to the misfolded collagens has been identified as a significant component of the disease pathology.^3^, ^4^

In the case of collagen I helix Gly substitutions, studies have shown that the mutations destabilize and delay helix formation and cause increased posttranslational modification of lysine and intracellular retention.^5^ However, the mutant collagens do not up‐regulate or bind to BiP, the sentinel chaperone recognizing misfolded proteins in the ER and the initiator of the canonical ER stress response.^6^ Subsequent studies on cells transfected with collagen I containing helix Gly mutations demonstrated that mutant‐containing collagen trimers aggregated in the ER and were degraded via autophagy.^7^ The precise nature of the ER stress response to collagen containing Gly substitutions is not yet clear and may involve mutation and chain (α1(I) or α2(I)) specificity.

Recent studies on a mouse model of mild to moderate OI (OI type IV) has yielded important information on disease mechanisms and possible therapeutic approaches.^8^ This model features a *Col1a2* triple helical codon 610 Gly to Cys substitution (α2(I) G610C), corresponding to a mutation first identified in an Amish family.^9^ The α2(I) G610C mutation disturbs the collagen triple helix and results, as expected, in ER accumulation of the mutant‐containing misfolded collagen trimers. This results in an unusual form of ER stress which does not involve the canonical unfolded protein response (UPR).^8^ This unconventional ER stress response involves modest up‐regulation of CHOP, eIF2α phosphorylation, and chaperones αβ crystalline and HSP47. It is also associated with striking negative effects on osteoblast function, affecting cell differentiation and maturation and an abnormal response to key signalling pathways. Importantly, this study showed that the ER retention of the mutant collagen stimulated autophagy and this was a key cellular adaptive response which modified the severity of the cell stress.^8^ Furthermore, they found that stimulating autophagy in osteoblasts in vitro with rapamycin reduced intracellular levels of mutant collagen and improved collagen secretion and extracellular matrix deposition.^8^ Thus, it is reasonable to hypothesize that therapeutic induction of autophagic degradation of the intracellularly retained mutant misfolded collagens should ameliorate the ER stress and deleterious downstream consequences on osteoblast differentiation and function in vivo. Preliminary testing of this approach used autophagy stimulation by a low‐protein diet in the OI mice.^10^ While this resulted in some beneficial effect on osteoblast differentiation and mineralization, the diet significantly reduced both WT and OI mouse growth, preventing any conclusions on the therapeutic effects on OI mouse bone.

To further explore the potential therapeutic benefit of autophagy induction in this α2(I) G610C OI mouse model, we treated mice with rapamycin, a well‐characterized autophagy stimulator.^11^, ^12^ Rapamycin is a commonly used immunosuppressant and chemotherapeutic drug which inhibits mTOR, a key nutrient sensitive serine‐threonine kinase. The mTOR pathway is involved promoting anabolic processes, ribosome biogenesis, protein synthesis and many cellular pathways, inhibiting cell stress responsive pathways, and protein degradation by autophagy.^13^ Inhibiting mTOR with agents such as rapamycin retards protein synthesis and enhances cell stress responsive pathways, such as autophagy.^11^ Treatment with rapamycin and rapalogs (rapamycin analogues), while mostly applied in cancer therapeutics, improves ER stress‐induced diabetes in mice^14^ and is generally beneficial in several mouse models of protein misfolding/aggregation neurodegenerative diseases such as Alzheimer's disease, Huntington's disease, Parkinson's disease, and amyotrophic lateral sclerosis.^11^ Rapamycin has also been shown to increase longevity of wild‐type mice.^15^ However in other studies, rapamycin was shown to exacerbate motor neuron degeneration in a *SOD1* mouse model of amyotrophic lateral sclerosis,^16^ suggesting caution must be taken when employing rapamycin (and rapalogs) and take into account the possible role of mTOR inhibition at the tissue level, disease, and mutation context. The role of mTOR signalling via the mTORC1 complex on osteoclast, osteoblast, and osteocyte differentiation and function is not fully understood with both positive and negative effects on bone formation reported.^17^ However, rapamycin is generally considered to be a largely bone‐sparing drug which may improve compromised bone quality.^18^, ^19^ In a recent study, mice with unloading induced bone loss were treated with rapamycin which restored osteoblast differentiation and bone volume.^20^ Similarly, rapamycin reduces the severity of senile osteoporosis in rats^21^ and osteopenia in mice with systemic sclerosis.^22^ Here we treat the α2(I)‐G610C OI mice with rapamycin for 5 weeks from weaning and measure the effect of this treatment on the structure and mechanical properties of the long bones and vertebrae to determine if rapamycin stimulation of autophagy is a viable new clinical approach in the treatment of OI caused by collagen I misfolding/aggregation mutations.

## 2 MATERIALS AND METHODS

### 2.1 Animals

Heterozygous α2(I)‐G610C osteogenesis imperfecta mice^9^ were obtained from the Jackson Laboratory, Bar Harbor, ME, USA (B6.129(FVB)‐Col1a2^tm1Mcbr/J^; stock number 007248) and maintained on a C57BL/6J background. Mice were housed 2‐5 animals/30 × 20 × 18 cm individually ventilated‐cage with filter lids, provided with sterilized bedding and environmental enrichment, maintained at 21‐22°C with a 12‐hour light/dark cycle, and water and complete pelleted food ad libitum. All procedures were approved by the Murdoch Children's Research Institute Animal Ethics Committee (Approval # A798). Dietary delivery of rapamycin was achieved using 154 ppm Eudragrit microencapsulated rapamycin (14 ppm active rapamycin) incorporated into Lab Diet 5LG6 (Test Diet, St. Louis, MO, USA) as previously described.^15^ The control 5LG6 diet contained 154 ppm Eudragrit. This rapamycin diet has been previously shown to result in detectable rapamycin in the blood and various mouse tissues^23^, ^24^ along with reduced mTORC signalling in several mouse tissues.^23^, ^25^ Male WT and α2(I)‐G610C mice were fed the rapamycin or control diets from 3 weeks of age until 8 weeks (n = 8 per experimental group). Mice were selected for the study by random allocation of male mice to control or rapamycin groups. There were no heterozygous runts and no mice were excluded. Regular serological assessment of sentinel mice was conducted to ensure the colonies were free of infection. Body weight was determined regularly during the 35 days of rapamycin treatment (daily determinations during the first week of treatment and three times a week for the remaining treatment period). At 8 weeks, mice were killed and tissues harvested for analysis.

### 2.2 Micro‐CT analysis

Ex vivo micro‐CT was performed on tibiae using the SkyScan 1076 system (Bruker‐micro‐CT) as previously described.^26^ Image acquisition settings were as follows: 9 μm voxel resolution, 0.5 mm aluminium filter, 48 kV voltage, 100 μA current, rotation 0.5°, frame averaging = 1. Images were reconstructed and analysed using SkyScan software programs NRecon (version 1.6.3.3), DataViewer (version 1.4.4), and CT Analyzer (version 1.12.0.0). Tibial trabecular analysis ROI was determined by identifying the tibia growth plate/primary spongiosa border and calculating a region that was 13.5% of total bone length, starting 4% away from the tibial growth plate. Trabecular bone structure evaluation was completed using adaptive thresholding in CT Analyzer, with a lower threshold limit of 0.18 mg/cm^3^ calcium hydroxyapatite (CaHA) respectively. Cortical analysis ROI was calculated by identifying a region that was 12% of tibial length, starting 40% away from the growth plate a lower global threshold of 0.35 mg/cm^3^ CaHA.

### 2.3 Mechanical testing

Right femora, right tibiae, and L4 vertebrae were frozen in saline‐soaked gauze at −80°C. After thawing, destructive testing was carried out by four‐point bending on an Instron 5944 mechanical testing machine (Canton, MA, USA) with data collected using Bluehill 3 software (Instron). Tibiae were positioned so that the medial side was resting across the supporting bottom span. Femora were positioned so that the anterior side was resting across the supporting bottom span. Bones were tested using a support span of 10 mm, with an upper span of 5 mm. Samples were pre‐loaded at 0.25 mm/min until a load of 1N was reached, at which point the loading rate increased to 0.5 mm/min until failure. Testing of vertebrae was performed on a custom jig featuring a support pin that threaded the neural canal to provide stability, and an upper plate with a corresponding female fit. The lower plate was covered with a fine grit sandpaper to minimize slippage. Prior to testing, vertebrae had their processes removed using scissors. Compression testing was carried out at 3 mm/min until failure with vertebrae oriented along their cephalocaudal axis, with the superior end facing up.^27^ Biomechanical properties were adjusted for body weight and bone length as described in the recent guidelines for mouse biomechanical analysis.^28^

### 2.4 Calvarial cell isolation and culture

Osteoblasts were obtained from the parietal bones of 10‐day‐old mice by sequential digestion with PBS containing 2 mg/mL collagenase (Worthington Biochemical Corporation; Collagenase II; Thermo‐Fisher) and containing 0.1% (w/v) trypsin.^29^ Digestion of the calvaria at 37°C for 20 minutes was repeated four times. The cells from the first two digestions (osteoblast progenitors) were discarded and cells from digestions 3 and 4 (osteoblasts) were pooled and expanded in DMEM (Gibco) containing 10% FBS (Gibco) at 37°C in a humidified atmosphere containing 5% CO_2_ and 95% air. At confluence, cells were stored frozen in aliquots of 1 × 10^6^ cells. Experiments were conducted on cells without passage or occasionally up to passage 2 to achieve sufficient cell numbers. Calvarial cells from WT and heterozygous α2(I)‐G610C mice were seeded at 2 × 10^4^ cells/cm and cultured in osteogenic medium DMEM, 10% FBS containing 50 μg/mL ascorbic acid 2‐phosphate (Sigma), and 10 mM β‐glycerophosphate (Sigma) for up to 21 days. Media was replaced every 2‐3 days.

### 2.5 Osteoblast apoptosis

At day 5 and 15 of culture in osteogenic medium, WT and OI calvarial cells were fixed in 4% PFA and apoptosis was assayed by TUNEL using the In Situ Cell Death Detection Kit, Fluorescein (Roche) according to the manufacturer's specification. WT calvarial cells treated with recombinant DNase I (Roche) for 30 minutes at room temperature prior to TUNEL staining were used as positive controls. Vectashield hard set antifade mounting medium with DAPI (Vector) was used as a mounting agent and a nuclear counterstain. Fluorescent microscopy was performed with an Axio Imager M1 fluorescence microscope (Zeiss). TUNEL‐positive nuclei were counted and expressed related to the total number of nuclei.

### 2.6 Mineralized matrix deposition

The ability of the osteoblasts to deposit calcium into the extracellular matrix was determined by Alizarin red dye staining. After 21 days, culture in osteogenic medium culture dishes were washed with PBS twice and then fixed with Confix Green (Pathtech) for 1 hour. The cells were washed with deionized water then stained with 2% (w/v) Alizarin Red S (Sigma Alrich) for 20 minutes. Cells were then washed with deionized water two times. The images of stained cells were captured using a digital camera.

### 2.7 Quantitative reverse transcription‐polymerase chain reaction (RT‐qPCR)

RNA was extracted from cells using the Trizol^®^ method (Qiagen) following the manufacturer's suggested protocol. RNA quantification was carried out using Nanodrop spectrophotometer (Thermo Scientific), and checked for RNA integrity by capillary electrophoresis with a Bioanalyzer 2100 (Agilent). About 1 μg of total RNA per sample was reverse transcribed using the Transcriptor High Fidelity cDNA Synthesis Kit (Roche) according to the manufacturer's instructions. For each sample, qPCR was performed in triplicate with the Brilliant III Ultra‐Fast SYBR Green QRT‐PCR Kit (Agilent). Gene‐specific primers were designed using Primer‐BLAST (NCBI) (Table S1). Amplification was performed in a 384‐well plate format on the LightCycler 480 Instrument II (Roche), using a thermocycling protocol involving initial denaturation at 95°C for 10 minutes, followed by 50 cycles of denaturation at 95°C for 30 seconds, annealing and polymerization at 60°C for 1 minute. Analyses of expression profiles were performed with LightCycler 480 Software (release 1.5.1.62). *Gapdh* was routinely used as a house‐keeping reference gene.

## 3 RESULTS

### 3.1 Rapamycin treatment inhibited growth selectively in OI mice

**Figure 1:**
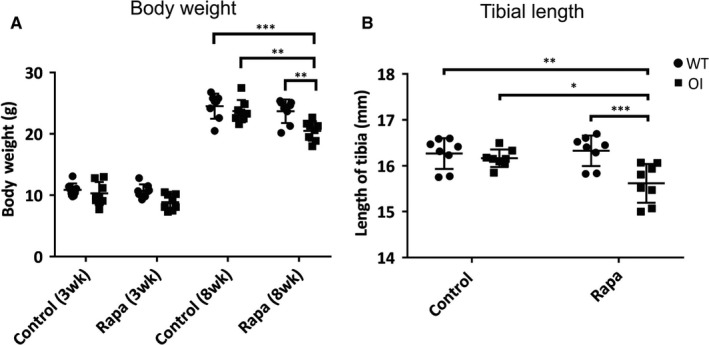
Effect of rapamycin treatment on mouse growth and long bone length. WT and OI male mice were treated with dietary rapamycin (Rapa) or control diet for 5 weeks from 3 weeks to 8 weeks of age. N = 8 mice per genotype and treatment. (A) Mouse body weight (g) at start of treatment and at end of 5 weeks treatment (+Rapa). Mean with 95% CI. (B) Tibial length at end of the treatment. Control and rapamycin (Rapa) treatment groups are indicated, ● WT, ■ OI. All values are the mean ± SD; Two‐way ANOVA with Tukey's post hoc test, *P < 0.05, **P < 0.01, ***P < 0.001. All WT and OI body weight data were significantly different (*P *< 0.0001) between 3 week and 8 week samples. For clarity in the figure, significance bars are not in shown between these samples

Osteoblasts isolated from OI and WT mice were used to confirm the cellular effects of the heterozygous *Col1a2* mutation on osteoblast function.^8^ Osteoblasts had increased *Ddit3* (Chop) mRNA expression (Figure S1A), increased apoptosis (Figure S1B), and showed a reduced capacity to deposit calcium in the extracellular matrix in vitro (Figure S1C). No change was seen in the expression of ER stress markers *Hspa5* (BiP) and *Atf4*, or in *Serpinh1* (Hsp47) in osteoblast culture (Figure 1A) as previously reported.^8^ While *Col1a1* mRNA levels were increased in OI osteoblasts (Figure S1D), there was a reduced expression of *Bglap* (osteocalcin), a marker of the mature osteoblast phenotype. *Runx2* expression also showed a trend towards reduced expression, although this was not statistically significant. These data support the conclusions that disrupted differentiation and malfunction of OI osteoblasts is caused by the intracellular accumulation of misfolded mutant procollagen triggering ER stress.

Previous studies have shown that OI mice have a slightly smaller body weight than WT mice at day 21 to day 60,^9^ and this trend was apparent in our OI mice (Figure S2) although the difference was not statistically significant at 3 weeks or 8 weeks of age (Figure 1A). Likewise, the length of tibiae (Figure 1B) was indistinguishable between OI and WT mice at 8 weeks. To test the effect of rapamycin on OI and WT bone parameters in vivo, 3‐week‐old male mice were administered rapamycin via their diet for 5 weeks until 8 weeks of age. Rapamycin treatment reduced the increase in body weight normally observed between 3 and 8 weeks in OI mice, but not in WT mice (Figure 1A). Similarly, OI but not WT, tibiae also showed impaired longitudinal growth after rapamycin treatment (Figure 1B). These data demonstrate that rapamycin treatment had a selective deleterious effect on the growth and skeletal development of OI mice.

### 3.2 Rapamycin increased trabecular bone mass in both WT and OI mice

**Figure 2:**
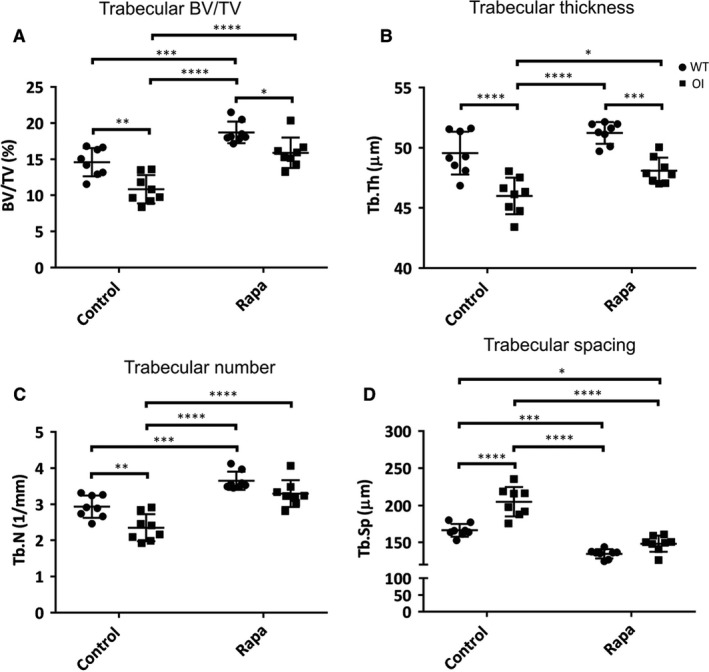
Micro‐CT of tibial trabecular bone. (A) BV/TV = bone volume fraction (%); (B) Tb.Th = trabecular thickness (μm); (C) Tb.N = trabecular number (mm^−1^); (D) Tb.Sp = trabecular separation (μm). Control and rapamycin (Rapa) treatment groups are indicated, ● WT, ■ OI. All values are the mean ± SD; Two‐way ANOVA with Tukey's post hoc test, *P < 0.05, **P < 0.01, ***P < 0.001, ****P < 0.0001

In WT mice, rapamycin led to a greater trabecular bone mass (Figure 2A), including greater trabecular number (Figure 2C) and lower trabecular separation (Figure 2D) compared to untreated controls. The OI mice had significantly lower trabecular bone mass (Figure 2A), trabecular thickness (Figure 2B), and trabecular number (Figure 2C), and greater trabecular separation (Figure 2D) than controls, as previously reported.^9^, ^30^ All these parameters were significantly improved in OI mice by rapamycin treatment (Figure 2). Moreover, they reached levels of trabecular bone mass (Figure 2A), thickness (Figure 2B), and number that were not significantly different from untreated WT trabecular bone. Rapamycin‐treated trabecular spacing was reduced compared to WT but similar to that of rapamycin‐treated WT mice. These data indicate that rapamycin treatment over this 5‐week developmental window may restore OI trabecular bone mass to levels comparable to WT trabecular bone.

### 3.3 Rapamycin treatment selectively reduced OI cortical bone mass

**Figure 3:**
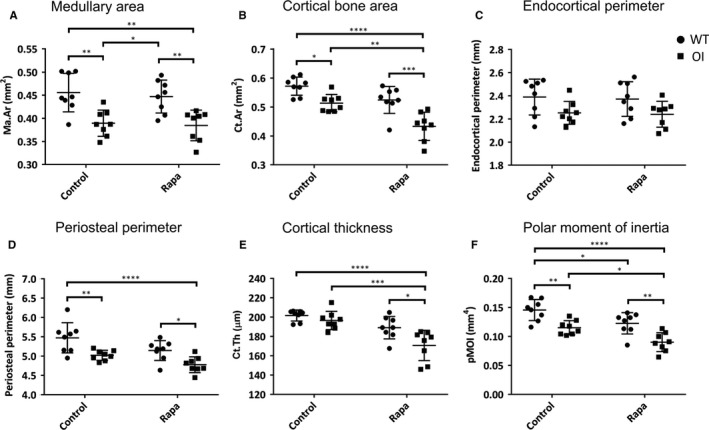
Micro‐CT of tibial cortical bone. (A) Ma.Ar = medullary area (mm^2^); (B) Ct.Ar = cortical bone area (mm^2^); (C) Endocortical perimeter (mm); (D) Periosteal perimeter (mm); (E) Cortical thickness (μm); (F) pMOI = polar moment of inertia (mm^4^). Control and rapamycin (Rapa) treatment groups are indicated, ● WT, ■ OI. All values are the mean ± SD; Two‐way ANOVA with Tukey's post hoc test, *P < 0.05, **P < 0.01, ***P < 0.001, ****P < 0.0001

In contrast to its effect on trabecular bone, rapamycin treatment for 5 weeks had no significant influence on the cortical bone micro‐CT parameters of WT mice (Figure 3A‐E) except the polar moment of inertia (Figure 3F). This showed a small but statistically significant reduction. Cortical bone from untreated OI mice showed significant deficits compared to WT bone in all cortical micro‐CT parameters (Figure 3A,B,D,F) except endocortical perimeter (Figure 3C) and cortical thickness (Figure 3E). Comparable data demonstrating the osteopenic phenotype of these OI mice have been previously reported.^9^, ^30^ Rapamycin treatment did not improve any of these OI cortical bone parameters, but to the contrary specifically further negatively impacted the OI cortical bone. The cortical area (Figure 3B), cortical thickness (Figure 3E), and polar moment of inertia (Figure 3F) were all significantly lower in rapamycin‐treated OI bones compared to untreated OI bones.

### 3.4 Mechanical deficiencies in OI long bone and vertebrae are not rescued by rapamycin

**Figure 4:**
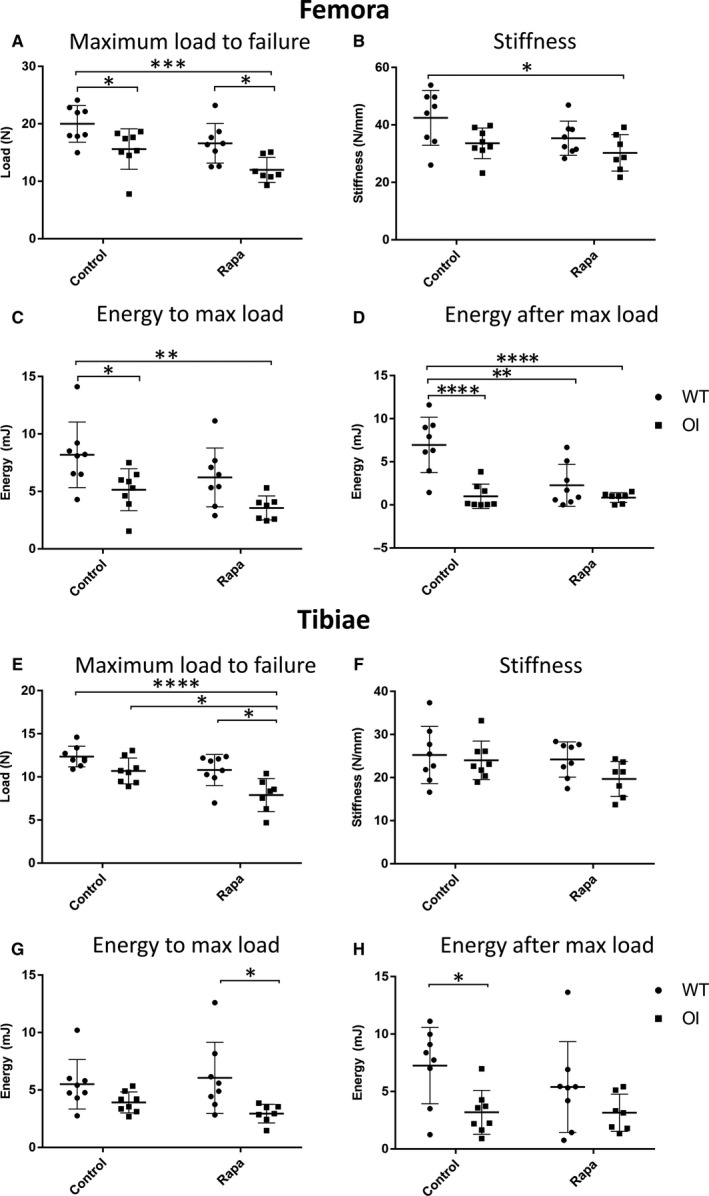
Four‐point bend testing of long bones. (A) Femora—Maximum load to failure (N); (B) Femora—Stiffness (N/mm); (C) Femora—Energy to maximum load (mJ); (D) Femora—Energy after maximum load (mJ); (E) Tibiae—Maximum load to failure (N); (F) Tibiae—Stiffness (N/mm); (G) Tibiae—Energy to maximum load (mJ); (H) Tibiae—Energy after maximum load (mJ). Control and rapamycin (Rapa) treatment groups are indicated; ● WT, ■ OI. Data were analysed using a two‐way ANOVA with Tukey's post hoc test. *P < 0.05, **P < 0.01, ***P < 0.001, ****P < 0.0001

**Figure 5:**
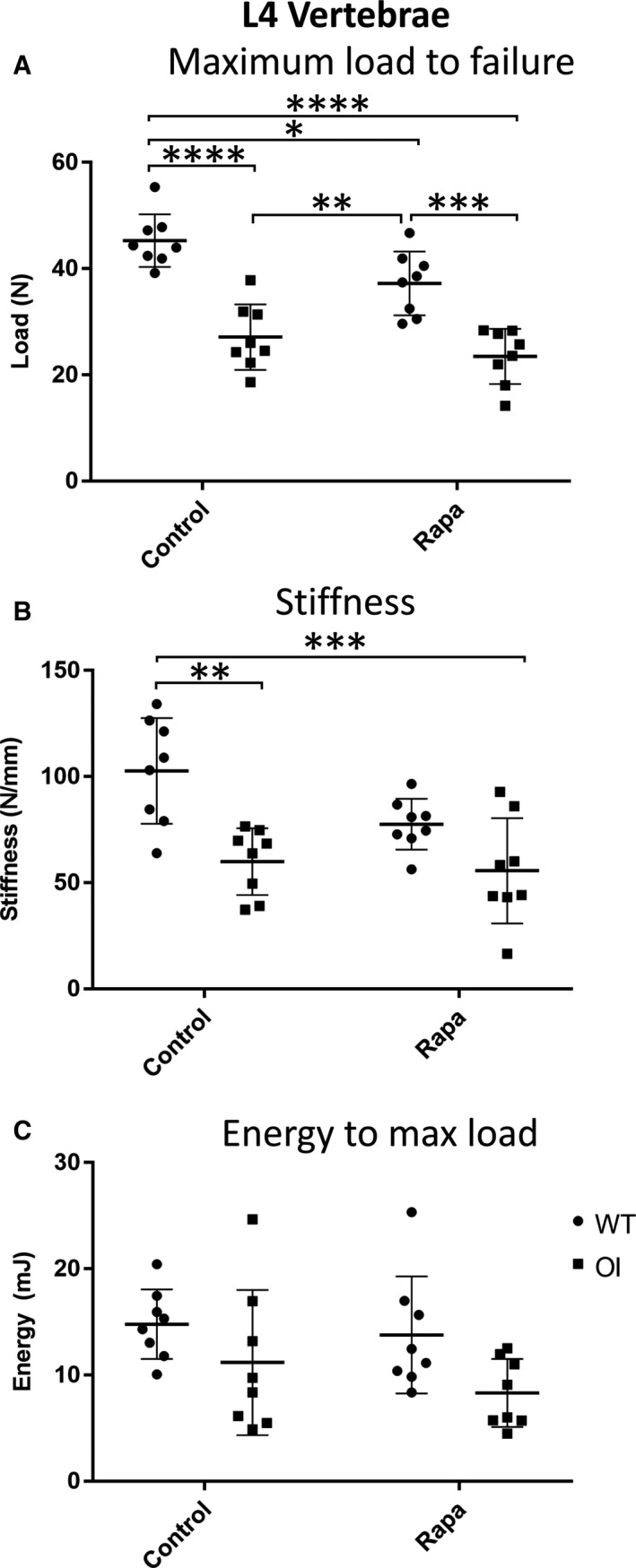
Compression testing of L4 vertebrae. (A) Maximum load to failure (N); (B) Stiffness (N/mm); (C) Energy to maximum load (mJ). Control and rapamycin (Rapa) treatment groups are indicated, ● WT, ■ OI. Data were analysed using a two‐way ANOVA with Tukey's post hoc test. *P < 0.05, **P < 0.01, ***P < 0.001, ****P < 0.0001

Biomechanical testing of WT and OI femora confirmed the biomechanical weakness of the OI long bones; they demonstrated reduced maximum load to failure and energy to maximum load (Figure 4A‐C). The most dramatic mechanical deficiency of untreated OI femora was revealed by analysis of energy after maximum load (Figure 5D) confirming the “brittleness” of the OI bones compared to untreated WT femora.^27^ Likewise, OI tibiae showed a comparable reduction in these parameters (Figure 4E‐H). Consistent with the negative impact of rapamycin on OI cortical bone parameters, the maximum load to failure for tibiae from rapamycin‐treated OI mice was further reduced (Figure 4E). Femora showed a similar trend (Figure 4E). In both femora and tibiae, the OI long bone brittleness (Figure 4D,H) was not rescued by rapamycin treatment. An important novel finding of our study was that rapamycin treatment of WT mice resulted in a reduced energy after maximum load of both femora and tibiae (Figure 4D,H) suggesting that this rapamycin treatment regime increases the brittleness of normal bones.

**Figure 6:**
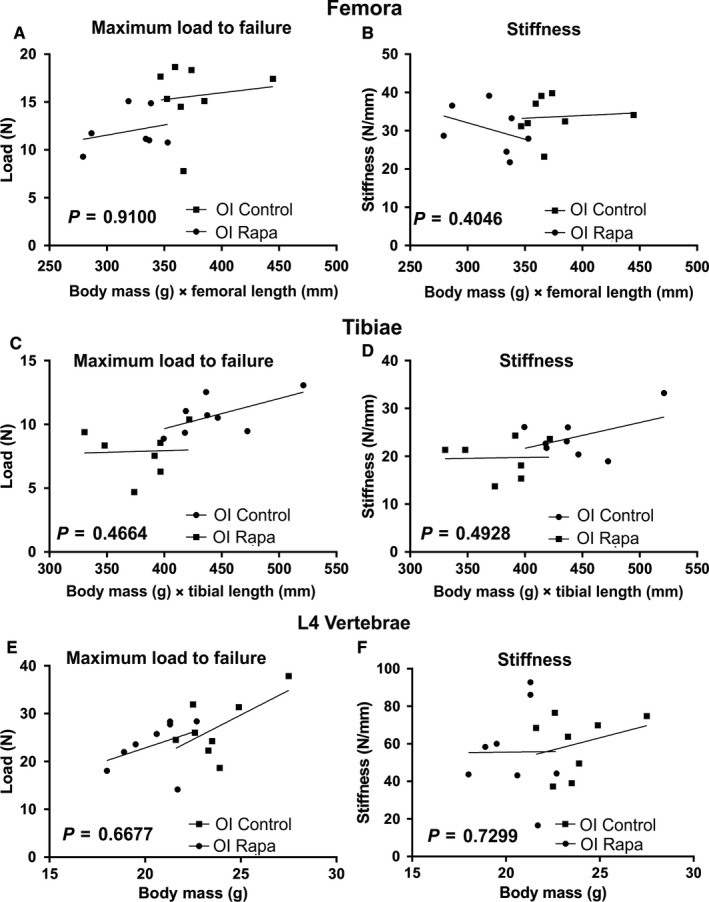
OI mechanical data corrected for body size and bone length. To correct for the reduced body weight and bone length of rapamycin‐treated OI mice the data were plotted against individual mouse weight × bone length previously described.^28^ For each plot, the significance (*P*‐value) of the difference between the slopes of untreated OI (■, OI Control) and rapamycin‐treated OI (●, OI Rapa) bones are indicated (ANCOVA). The *r* ^2^ value and *P*‐value for each plot is given below to describe if the slope is significantly non‐zero. (A) Femora—Maximum load to failure (N); OI Control *r* ^2^ = 0.01558, *P* = 0.7684; OI Rapa *r* ^2^ = 0.07437, *P* = 0.5540. (B) Femora—Stiffness (N/mm); OI Control *r* ^2^ = 0.00668, *P* = 0.8475; OI Rapa *r* ^2^ = 0.1436, *P* = 0.4019. (C) Tibiae—Maximum load to failure (N); OI Control *r* ^2^ = 0.3598, *P* = 0.1160; OI Rapa *r* ^2^ = 0.00185, *P* = 0.9271. (D) Tibiae—Stiffness (N/mm): OI Control *r* ^2^ = 0.2116, *P* = 0.2514; OI Rapa *r* ^2^ = 0.00069, *P* = 0.9554. Compression testing of L4 vertebrae. (E) Maximum load to failure (N); OI Control *r* ^2^ = 0.365, *P* = 0.1127; OI Rapa *r* ^2^ = 0.1602, *P* = 0.3259. (F) Stiffness (N/mm); OI Control *r* ^2^ = 0.08933, *P* = 0.4721; OI Rapa *r* ^2^ = 0.00005, *P* = 0.9870

As rapamycin‐treated OI mice had lower body weight and bone length compared to WT and untreated OI mice (Figure 1), we normalized the whole bone mechanical data to account for this in case the apparent biomechanical deficiencies reflected the reduced size of the OI bones.^28^ These data are shown in Figure 6A‐D. In femora, the slopes of the lines comparing the maximum load to failure (Figure 6A) and stiffness (Figure 6B) against body mass × bone length^28^ were not significantly different (*P* = 0.9100 and *P* = 0.4046 respectively), demonstrating that bone mechanical properties scale with mouse size rather than with rapamycin treatment. Similar results were obtained (*P* = 0.466 for maximum load to failure; *P* = 0.5119 for stiffness) when tibial biomechanical parameters were adjusted for the reduced size of the rapamycin‐treated OI mice (Figure 6C,D).

More dramatic deficits in the OI bone material properties were observed by vertebral compression testing (Figure 5A‐C). Both maximum load to failure (Figure 5A) and stiffness (Figure 5B) of L4 vertebrae were significantly reduced in OI mice compared to WT mice. The OI mouse L4 vertebral mechanical deficits were not improved by rapamycin treatment (Figure 5A‐C) even when the smaller size of the rapamycin‐treated OI mice was accounted for by plotting individual mouse weights against the mechanical parameters (Figure 6E,F).

## 4 DISCUSSION

Surgical management of skeletal abnormalities and bisphosphonate treatment to reduce bone catabolism are the primary methods for the clinical care of OI patients. Bisphosphonate therapy has been widely adopted and is effective in improving bone mass and micro‐architecture with a consequent small reduction in fracture rate in OI patients. While bisphosphonates offer a therapeutic approach, they do not resolve the fundamental issue of reduced bone quality such as that caused by collagen structural mutations. Collagen I mutations, including the common OI collagen helix Gly mutations, cause misfolding and ER collagen retention in collagen I‐producing cells such as osteoblasts.^3^ The formation of these toxic intracellular protein aggregates activates a poorly characterized ER stress response with detrimental cellular consequences impacting bone formation and quality. This suggests that stimulating proteolysis to reduce this intracellular misfolded protein load could be an important alternative (or complementary) approach to OI therapy.

As proof‐of‐principle of this approach, we used a mouse model of mild‐moderate OI, caused by a heterozygous collagen I helix‐disrupting mutation, α2(I)‐G610C. This OI preclinical model has been previously used to study the therapeutic effects of sclerostin antibody treatment,^31^ and combination therapy of sclerostin antibody and bisphosphonate.^27^ Baseline characterization of osteoblasts cultured from this mouse model confirmed that the mutation resulted in an unconventional ER stress response with downstream signalling consequences. This included up‐regulation of *Chop* and increased apoptosis, along with altered mouse calvarial osteoblast differentiation in vitro.^8^ While determining the precise consequences of the misfolded collagen helix on ER stress and degradation pathways will be important to understand the molecular pathology of OI mutations, the key finding was that activation of autophagy by rapamycin reduced mutant collagen accumulation in the ER and improved the ability of the OI cell in vitro to deposit a collagen matrix.^8^ This suggested that autophagy activation may offer a useful therapeutic strategy in vivo.

The capacity of dietary rapamycin to rescue the OI phenotype was tested by treating male OI and WT mice for 5 weeks, during a period of high juvenile bone growth from weaning until 8 weeks of age. Despite its many potential cellular effects, rapamycin is clinically well‐tolerated and has been used effectively in several studies on protein misfolding/aggregation disorders.^11^ Rapamycin‐treated WT mice had normal body weight and long bone length, and the improvement in trabecular bone structure with rapamycin treatment is consistent with the concept that an autophagy‐inducing low‐protein diet may improve osteoblast differentiation and/or bone mineralization.^10^ Recent studies have similarly shown that autophagy promotes osteogenic differentiation of human bone marrow mesenchymal stem cells^32^ and osteoblast‐specific ablation of autophagy in mice results in reduced bone mass.^33^ Moreover, in several mouse or rat models of osteopenia, rapamycin treatment was beneficial,^20^, ^22^ suggesting possible therapeutic applications in some bone loss conditions. In addition, it has been reported that rapamycin‐induced autophagy improves bone fracture healing.^34^, ^35^

In contrast to the beneficial effects on the trabecular compartment of WT long bones, rapamycin treatment had an apparent negative impact on cortical bone in WT mice. There was a small reduction in the polar moment of inertia and biomechanical testing demonstrated a reduced energy after maximum load reflecting increased WT bone brittleness. Perhaps linked to the reduced polar moment of inertia, this novel finding of an increased brittleness of rapamycin‐treated WT mouse cortical bone is in contrast to the previous studies that have suggested rapamycin is bone‐sparing.^18^, ^19^, ^21^ Rapamycin had no significant impact on the other mechanical properties of the WT mice femora or tibiae such as maximum load to failure, stiffness, or energy to maximum load. In mechanical testing of the WT vertebrae, there were reductions both in stiffness and maximum load to failure after rapamycin treatment. Our studies, which show for the first time that rapamycin can have some negative impacts on normal bone, indicate that additional studies are required to refine our understanding of rapamycin and other mTOR‐targeted therapies on the mechanical strength of WT bone. It will be important to determine if these impacts are of clinical consequence and outweigh the positive effects on trabecular bone in considering therapies targeting mTOR signalling and/or autophagy in treating osteoporotic conditions.

The positive effects of rapamycin on the OI trabecular bone micro‐CT outcome were striking, increasing all the trabecular structural parameters to those of untreated WT bones. In contrast, rapamycin reduced periosteal growth in OI bone, leading to reduced cortical area, thickness, and moment of inertia. Likely because of this reduction in moment of inertia, rapamycin did not improve the biomechanical properties of OI femora or tibiae. In fact, at first glance, our data seem to suggest that rapamycin actually further reduced OI bone biomechanics. However, when we corrected for the deleterious effect of rapamycin on body weight and long bone growth in the OI mice, it was apparent that the maximum load to failure of treated and untreated mouse femora, tibiae, and vertebrae were not significantly different. Vertebral strength was also not improved in the OI mice treated with rapamycin. Thus, we conclude that the reduced mechanical strength of rapamycin‐treated OI mouse bones predominantly reflects the suppression of both longitudinal and transverse bone growth in OI mice. These results showing that rapamycin selectively inhibits OI bone growth during this active growth phase is an important finding, clearly indicating that rapamycin may have a deleterious effect on bone growth in OI patients with collagen I misfolding mutations. A limitation of our study was that it was conducted on only male mice. While there is no evidence to suggest that female mice will respond differently, it will be nonetheless important to confirm these rapamycin results in female mice.

The molecular basis of this selective impact on the growth of the α2(I)‐G610C mutant mouse bone is not known. Rapamycin acts by associating with FK506‐binding protein 12 which then binds the mTOR Complex 1 (mTORC1) preventing downstream signalling. While autophagy stimulation is a key downstream consequence, it is important to appreciate that mTORC1 is a signal integrator that is involved in numerous cellular processes involved in cell growth and energy metabolism, including protein synthesis.^13^ The role of mTOR signalling in bone formation and homeostasis is still controversial with evidence for both stimulation and inhibition of osteogenic differentiation and bone formation.^17^, ^36^, ^37^, ^38^, ^39^, ^40^, ^41^ In this OI mouse model, a major impact of the ER stress induced by collagen I misfolding and aggregation is altered osteoblast differentiation and maturation. As a result, the OI osteoblasts may have a different response to rapamycin exposure than WT osteoblasts and this may contribute to reduced bone growth. It is also possible that rapamycin could also affect OI chondrocyte hypertrophy and this could contribute to the reduced endochondral bone growth in the OI mice. Unlike proliferating chondrocytes, the growth plate cartilage hypertrophic chondrocytes also synthesize collagen I. As a result, the OI hypertrophic chondrocytes may also be subject to some ER stress as a result of the intracellular retention of the misfolded mutant OI collagen I. Such ER stress caused by collagen misfolding has been shown to disturb key signalling pathways^42^, ^43^ and this may render the OI hypertrophic chondrocytes susceptible to rapamycin treatment. The coupling of mTORC1 signalling to ER stress and proteostasis^44^ highlights the possibility that the OI mouse ER‐stressed osteoblasts, osteocytes, and possibly ER‐stressed chondrocytes, could be selectively vulnerable to mTOR inhibitors resulting in the suppression of OI bone growth. The differential effects of rapamycin on OI trabecular and cortical bone compartments raise a number of important questions. Is the deposition, turnover, and pathological impact of the mutant collagen similar in these two compartments? We have previously shown in long term in vitro matrix formation by OI fibroblasts that not only is total collagen deposition poor but also that the mutant collagen was unstable and degraded over time. This suggested that the mutant collagen was either enriched on the exposed surfaces of WT/mutant composite collagen fibers, or polymerized into mutant‐only fibrils prone to proteolytic attack.^45^ Thus, it is possible that the amount of mutant, its organization, and structural consequences may be affected by differential bone remodelling in trabecular vs cortical bone. Likewise, the extent of ER stress and its downstream consequences may be subtly different in trabecular and cortical bone compartments.

This study cannot distinguish between effects of altered mTOR signalling and autophagy. To evaluate the true therapeutic value of autophagy stimulation in OI resulting from collagen misfolding mutations, it will be important to test other autophagy modulators with increased specificity,^12^ and in particular those independent of the mTOR pathway. Further impetus for these studies is provided by recent data showing that stimulation of intracellular proteolysis by carbamazepine ameliorated the pathological impact of mutant collagen X‐induced growth plate cartilage ER stress in a mouse model of metaphyseal chondrodysplasia.^46^ While the mutant collagen X was degraded by carbamazepine‐induced autophagy in the mouse model, in vitro studies with other collagen X mutations showed that carbamazepine could reduce accumulated intracellular mutant by both autophagy and proteasomal pathways.^46^ These data provide support for further testing the therapeutic benefit of stimulating the autophagy and/or proteasomal pathways to reduce the intracellular accumulation and secretion of toxic misfolded proteins in OI and other skeletal dysplasias.

We conclude that the interaction of rapamycin with bone in the context of OI is complex and may be fundamentally altered compared to healthy bone. We show that rapamycin treatment offered no functional benefit in this OI preclinical model, and suggest that the impact of rapamycin on OI bone growth could potentially exacerbate the clinical consequences in OI patients during periods of active bone growth. These data underline the need for clinical caution about using mTOR inhibitors, rapamycin, and rapalogs, in patients with OI caused by collagen mutations.

## CONFLICTS OF INTEREST

The authors declare no conflicts of interest.

## Supporting information


**Data S1**. Cell stress, apoptosis, and differentiation in cultured parietal bone osteoblasts.
