# Supplementary material for: Effect of rapamycin on bone mass and strength in the α2(I)‐G610C mouse model of osteogenesis imperfecta
Source: J Cell Mol Med. 2018 Dec 30;23(3):1735–45. doi: 10.1111/jcmm.14072 (PMC6378195; doi:10.1111/jcmm.14072)
Supplement: Supplementary file 1 — Data S1. Cell stress, apoptosis, and differentiation in cultured parietal bone osteoblasts. [file JCMM-23-1735-s001.pdf]

Supplementary Data

Effect of rapamycin on bone mass and strength in the α2(I)-G610C mouse model of osteogenesis imperfecta

Bateman et al

Supplementary Table 1

RT-qPCR primer list

**Gene**	**Forward primer**	**Reverse Primer**
Gapdh	AATGTGTCCGTCGTGGATCT	GCTTCACCACCTTCTTGATGT
Hspa5 (BiP)	TGAAACTGTGGGAGGAGTCA	TTCAGCTGTCACTCGGAGAA
Ddit3 (Chop)	GCGACAGAGCCAGAATAACA	GATGCACTTCCTTCTGGAACA
Serpinh1 (Hsp47)	ATGCCTGCCCTAGAATTGTTAT	AAAAGGGAGAAAAACAAAAGTTCA
Atf4	AGAAGCTGAAAAAGATGGAGCA	GCTTCTTCTGGCGGTACCTA
Col1a1	GACTGGCAACCTCAAGAAGG	CAAGTTCCGGTGTGACTCG
Runx2	TCTCCACACCATTAGAGGTTGA	GGGTGCTTCTGCTACCACTC
Bglap	AGACTCCGGCGCTACCTT	CTCGTCACAAGCAGGGTTAAG

Supplementary Figure 1. Cell stress, apoptosis and differentiation in cultured parietal bone osteoblasts

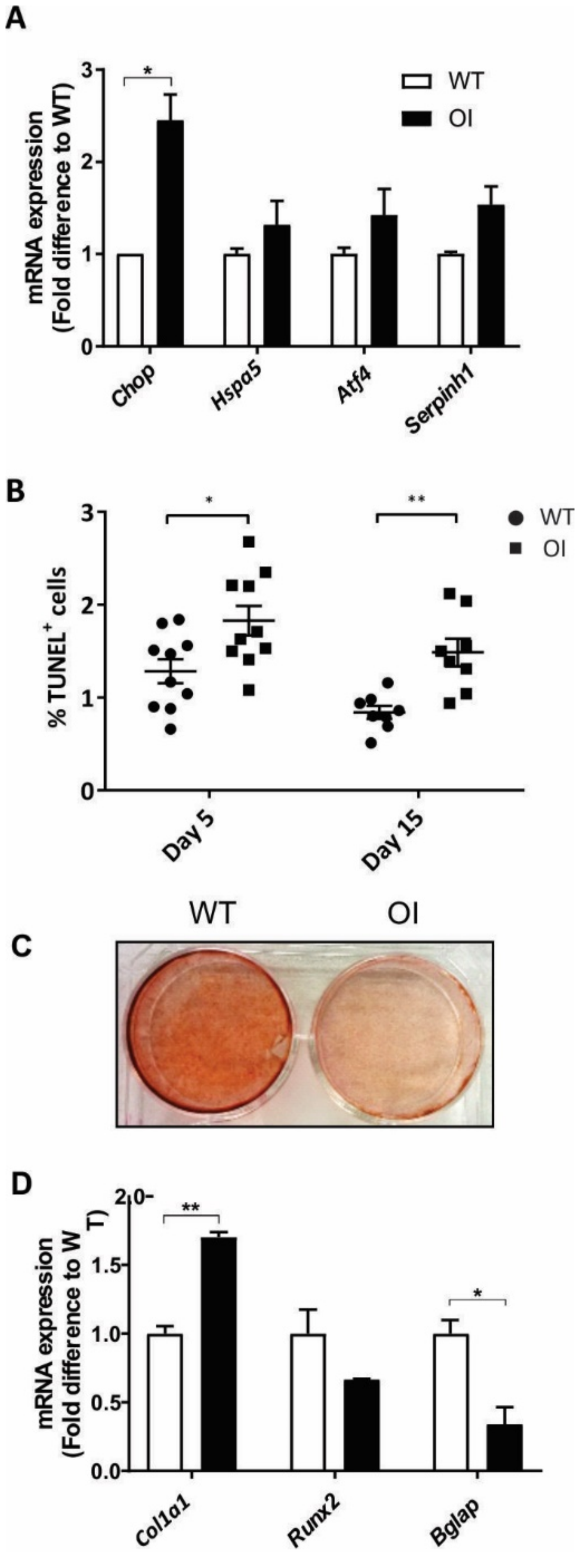

A) Primary osteoblasts from 10 day old wild type (WT) and OI mice were cultured for 21 days in osteogenic medium, mRNA extracted and RT-qPCR performed in triplicate using primer pairs for the indicated genes normalised to the housekeeping gene *Gapdh*. Plots depict mean fold difference of OI to WT control with standard deviation from the mean, N = 3 independent experiments, statistical significance was determined assuming a normal distribution among the groups using unpaired one-tail Student's *t* test,* $p < 0.05$; B), Apoptosis in calvarial osteoblasts. Calvarial cells were cultured *in vitro* in osteogenic medium, for 5 and 15 days, fixed and apoptosis was assayed by TUNEL staining. TUNEL-positive nuclei were counted and expressed related to the total number of DAPI-positive nuclei. □ WT, ■ OI. All values are mean ± S.E.M. Student's t-test, * $p < 0.05$, ** $p < 0.01$; **C**), Cultures stained with Alizarin Red to visualize calcification after 21 days in osteogenic medium; **D**), RT-qPCR of osteoblast differentiation markers after 21 days culture as described in A, * $p < 0.05$, ** $p < 0.01$.

Supplementary Figure 2. OI and WT mouse weight

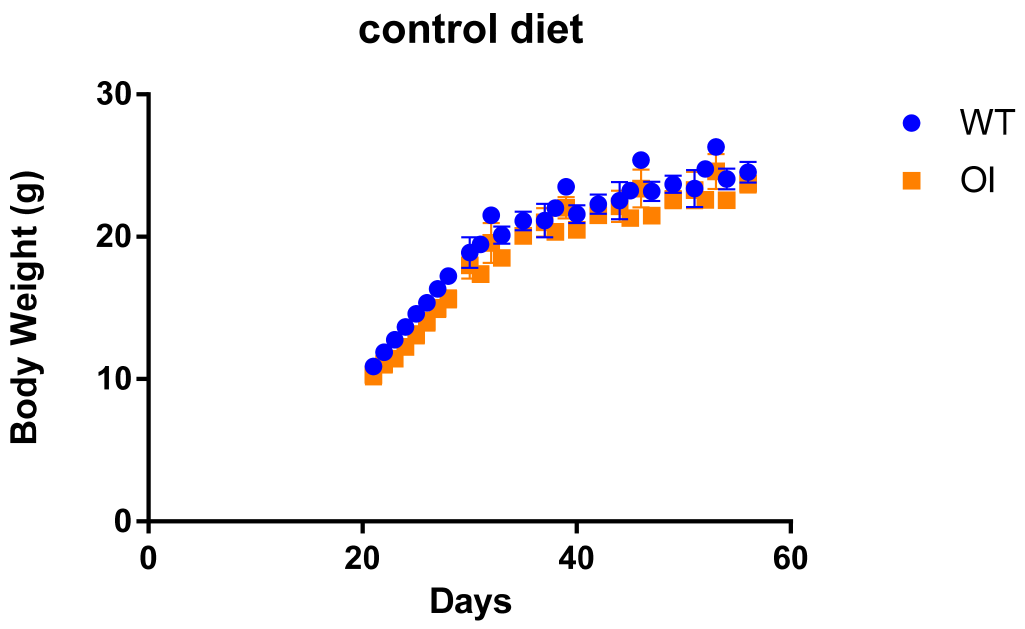

Wild type mice (N=8) and OI mice (N=8) were weighed daily from 3 weeks to 8 weeks of age.

All values are mean ± S.E.M.
